# Sleep disturbances and their relationship with anxiety–depressive symptoms and quality of life in older adults: a retrospective observational study

**DOI:** 10.1515/med-2026-1491

**Published:** 2026-07-20

**Authors:** Huiqing Xuan, Lixiu Wu, Kunqiang Yu, Lu Yang, Xiaoyan Chen

**Affiliations:** Department of Psychiatric Rehabilitation, The Second People’s Hospital of Lishui, Lishui, Zhejiang Province, China; Central Laboratory, The Second People’s Hospital of Lishui, Lishui, Zhejiang Province, China; Department of Psychiatry, The Second People’s Hospital of Lishui, Lishui, Zhejiang Province, China; Department of Neurology, The Second People’s Hospital of Lishui, Lishui, Zhejiang Province, China

**Keywords:** sleep disorders, aged, anxiety, depression, quality of life

## Abstract

**Objectives:**

To investigate the prevalence of sleep disorders in the elderly and explore their relationships with anxiety, depressive symptoms, and quality of life.

**Methods:**

This single-center retrospective study included 120 elderly inpatients from the psychiatry department of our hospital between December 2020 and December 2025. Demographic and clinical data, along with Pittsburgh Sleep Quality Index (PSQI), Hospital Anxiety and Depression Scale (HADS), Patient Health Questionnaire-9 (PHQ-9), 12-Item Short Form Health Survey (SF-12) scores and the Chinese version of the Montreal Cognitive Assessment Scale (MoCA-C), were collected. Patients were categorized into sleep disturbance (SD, PSQI>5) and non-sleep disturbance (NSD, PSQI≤5) groups. Group differences were analyzed, and multivariate logistic regression and Spearman correlation were performed.

**Results:**

Of the 120 patients, 50 (41.7 %) had sleep disturbances. The SD group showed significantly higher anxiety and depression scores and lower SF-12 and MoCA scores than the NSD group (all p<0.05). The total PSQI score was strongly positively correlated with HADS anxiety, HADS depression, and PHQ-9 scores (r=0.413–0.514, p<0.001), and significantly negatively correlated with SF-12 PCS, SF-12 MCS, and MoCA-C scores (r=−0.683 to −0.476, p<0.001). Multivariate logistic regression identified chronic pain (OR=3.580, 95 % CI: 1.444–8.879, p=0.006), long-term sedative-hypnotic use (OR=2.860, 95 % CI: 1.136–7.200, p=0.026), and psychological loneliness (OR=5.261, 95 % CI: 2.006–13.798, p=0.001) as independent risk factors for sleep disorders in the elderly.

**Conclusions:**

In this hospital-based sample of older psychiatric patients, sleep disturbances in older adults are significantly associated with anxiety, depression, and reduced quality of life, particularly mental health. Early screening and targeted interventions are warranted.

## Introduction

Sleep is a core biological process that maintains the body’s physiological functions, cognitive integration, and emotional regulation. With advancing age, sleep structure changes significantly, manifested as reduced deep sleep, increased sleep fragmentation, more frequent nocturnal awakenings and disrupted circadian rhythms [1], [2], [3]. These age-related physiological changes, together with comorbid chronic diseases, polypharmacy and social environmental shifts, make the elderly a high-risk group for sleep disorders [4], 5]. Sleep disorders have multifaceted effects on both the physical and psychological well-being of elderly individuals. At the physiological level, sleep disorders may trigger or aggravate anxiety and depressive symptoms via hypothalamic-pituitary-adrenal (HPA) axis dysfunction and monoamine neurotransmitter imbalance [6], and are closely linked to adverse outcomes including cardiovascular diseases, cognitive decline, immune dysfunction and elevated fall risk [7]. Chronic low-grade inflammation is regarded as one of the core mechanisms of multiple age-related diseases in the elderly, involving immune and neuroendocrine disorders [8]. At the psychological level, sleep disorders have a complex bidirectional relationship with anxiety and depressive symptoms. A longitudinal network analysis in Chinese elderly individuals showed that poor sleep quality had the strongest bridging edge with tension/anxiety, indicating that sleep problems act as a core node connecting depressive and anxiety symptom clusters [9].

Sleep physiology research demonstrates that normal human sleep architecture undergoes substantial remodeling with advancing age. Such age-related sleep alterations are mainly characterized by shortened total sleep time, reduced sleep efficiency, marked decrease or even disappearance of slow wave sleep, and prolonged wake after sleep onset [3]. In the elderly, the deterioration of the circadian rhythm system (e.g., reduced neurons in the hypothalamic suprachiasmatic nucleus and an earlier, lower melatonin secretion peak) causes shifts in the sleep-wake cycle, which are often manifested as advanced bedtime and wake-up time, as well as sleep fragmentation during the night [10]. Although some changes in sleep architecture are part of normal physiological aging, they constitute clinically significant sleep disorders when accompanied by daytime dysfunction, cognitive decline, or emotional distress. Previous epidemiological surveys have reported a prevalence of sleep disorders of approximately 30–45 % among community-dwelling older adults, which can exceed 60 % in hospitalized elderly individuals or those with chronic physical diseases [11].

When exploring the clinical impacts of sleep disorders in the elderly, anxiety and depression are two indispensable comorbid factors. Late-life depression is not only manifested as persistent low mood but also often accompanied by severe somatic symptoms, cognitive impairment, and suicidal ideation; while late-life anxiety is mainly characterized by excessive worries about one’s health, financial status, and family relationships [12]. Recent psychoneuroendocrinological studies strongly suggest that sleep disorders and anxiety/depressive symptoms do not follow a one-way causal relationship but instead form a bidirectional vicious cycle pattern based on complex biological mechanisms [12]. On one hand, due to metabolic abnormalities of monoamine neurotransmitters (e.g., serotonin, norepinephrine) in the central nervous system, depression and anxiety themselves disrupt sleep continuity. On the other hand, chronic sleep deprivation or fragmentation leads to overactivation of the HPA axis, abnormally elevated cortisol levels, and systemic low-grade inflammation, thereby inducing or exacerbating depressive and anxious emotions [13]. Although this bidirectional mechanism has been partially verified theoretically, the specific interaction between sleep disorders and anxiety or depressive symptoms still requires quantitative evaluation with systematic clinical data. This is particularly necessary for the elderly with multiple underlying diseases and polypharmacy in real clinical settings.

Based on the above research background and clinical dilemmas, this study puts forward the following hypotheses: sleep disorders occur at a high rate in geriatric individuals, and sleep disorders serve as independent risk factors that exacerbate symptoms of anxiety and depression and compromise overall quality of life. Therefore, the purpose of this study is to retrospectively collect and analyze the detailed clinical and follow-up data of elderly patients treated in a single center during the recent five years (2020–2025), so as to objectively evaluate the incidence and clinical characteristics of sleep disorders in the elderly. Through comparing the scores of various standardized psychological and quality-of-life scales between elderly patients with sleep disorders and those without, this study intends to quantitatively explore the intrinsic correlation between sleep and psychological status. To exclude confounding factors and determine independent risk factors for sleep disorders in older adults, multivariate Logistic regression models will be applied. Through multidimensional data mining, this study expects to provide detailed, reliable and instructive scientific evidence for clinicians in the early identification and precise intervention of sleep problems in the elderly, as well as in improving their mental and psychological prognosis.

## Methods

### Research subject

**Figure 1:**
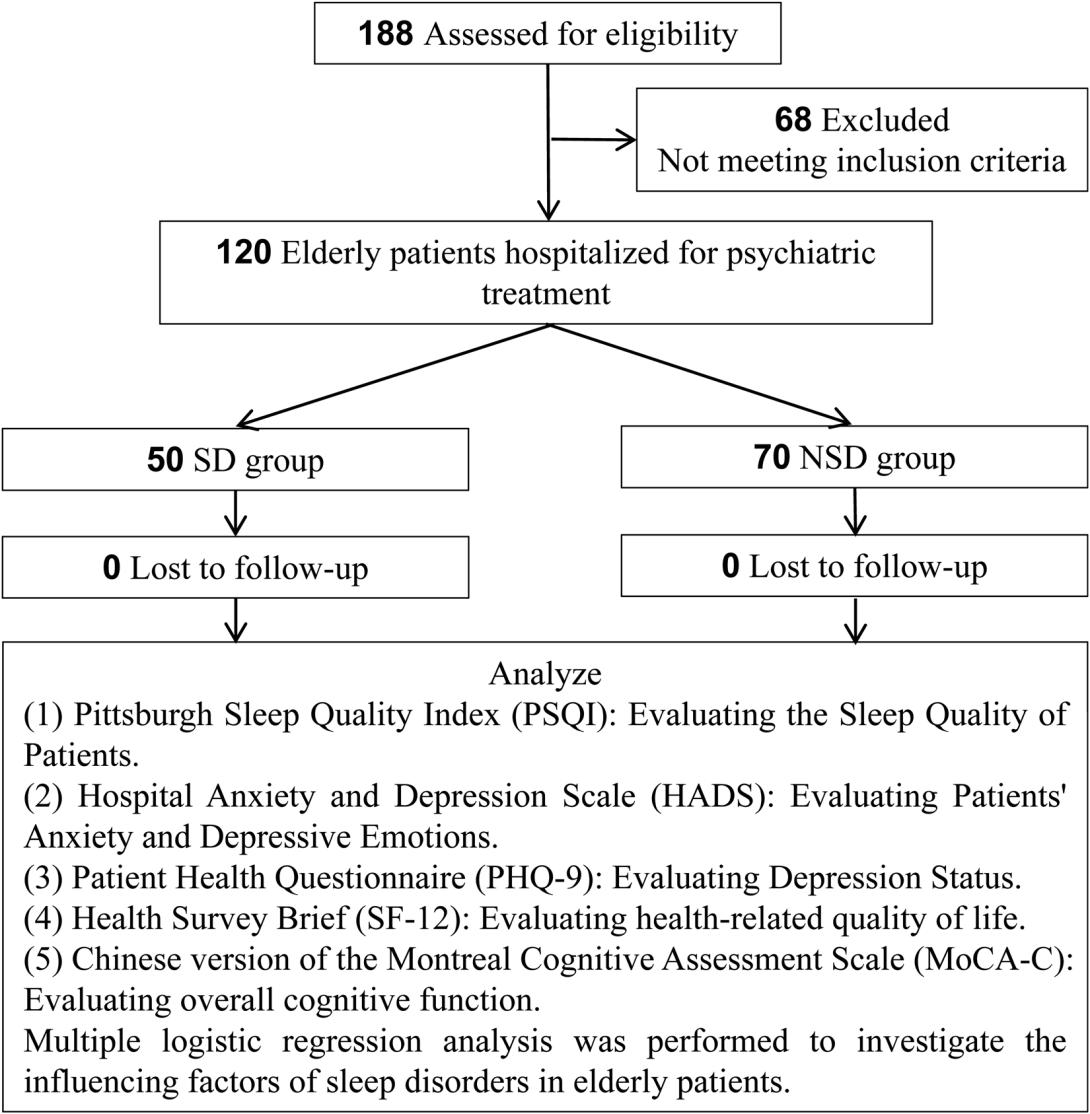
Research flowchart.

This single-center retrospective observational study consecutively included elderly inpatients from our hospital’s psychiatry department (Dec 2020–Dec 2025) via electronic medical records. Demographic, clinical data and Pittsburgh Sleep Quality Index (PSQI) scores were collected. Patients were grouped into SD (PSQI>5) and NSD (PSQI≤5) groups. Intergroup differences in anxiety, depression and quality of life were compared, and their correlations analyzed. The patient selection process is illustrated in Figure 1.

### Inclusion criteria

(1) Age ≥60 years old;
(2) Complete medical records were available, including demographic characteristics, medical history, and major medication history;
(3) Basic normal cognitive function was preserved, with the ability to read, understand, and complete standardized questionnaire surveys independently (or with the assistance of family members);
(4) Complete scale assessment results such as the PSQI, Hospital Anxiety and Depression Scale (HADS), Patient Health Questionnaire (PHQ-9), Short Form Health Survey (SF-12) and the Chinese version of the Montreal Cognitive Assessment Scale (MoCA-C) were recorded during the hospitalization period or in the follow-up files.

### Exclusion criteria

(1) Sleep abnormalities secondary to severe mental illnesses such as severe schizophrenia and acute phase of bipolar disorder;
(2) Presence of severe neurological diseases (e.g., advanced Alzheimer’s disease, disturbance of consciousness caused by sequelae of acute stroke);
(3) Complicated with end-stage diseases such as advanced malignant tumor and severe organ failure with a life expectancy of less than 6 months;
(4) Cases with a missing rate of clinical and scale data >5 %.

### Ethical statement

Approval for this study protocol was granted by The Second People’s Hospital of Lishui Ethics Committee (No. 2025-024). All data collection and retrospective analysis procedures met the standards specified in the Declaration of Helsinki [14]. This study used anonymous retrospective medical record analysis, and no direct contact was made with the subjects. All identifiable personal information was de-identified to safeguard data confidentiality.

### Sample size calculation

The sample size estimation for this study was conducted using G * Power 3.1 software [15]. Previous literature reports have shown that the overall effect size of the association between sleep disorders and depressive symptoms in the elderly population is relatively small (beta=0.166, Cohen’s f^2^ is a small effect) [16], while the intergroup difference effect size of sleep disorders on quality of life is about moderate (Cohen’s d=0.35–0.61) [17]. *A priori* sample size was estimated for an independent-samples t-test with a two-sided α=0.05 and power=0.80. Based on a medium effect size of Cohen’s d=0.6, the required sample size was 44 patients per group, totaling 88 patients. Considering a 10 % potential dropout or incomplete data rate, we aimed to enroll at least 97 patients. The final sample of 120 patients exceeds this requirement.

### Data collection and grouping

Two uniformly trained clinical investigators retrospectively extracted patient data in a double-blind manner from the hospital electronic medical record system and the psychological assessment archive.

The collected data included:

General demographic data: age, sex, body mass index (BMI), marital status, educational level, and the presence of persistent loneliness complaints (based on psychological assessment records in medical charts).

Clinical characteristics: number of comorbid chronic conditions, smoking history, drinking history, and long-term use of sedative-hypnotic drugs (continuous use for >3 months).

According to the original validation study by Buysse et al. [18], the developers of the PSQI, a PSQI score >5 (≥6) was recommended as the cutoff to distinguish good and poor sleep quality, with favorable sensitivity of 0.896 and specificity of 0.865 [19]. Therefore, in this study, PSQI>5 points were defined as the sleep disorder group (SD group), and PSQI≤5 points were defined as the non sleep disorder group (NSD group).

### Evaluation tool

(1) PSQI [18], 20]: This scale assesses past-month sleep quality across 7 domains (0–3 each; total 0–21). Higher scores indicate poorer sleep.
(2) HADS [21], 22]: This scale screens anxiety and depression in general hospital patients, reducing physical disease confounders. It has two 7-item subscales (max 21 points each); higher scores mean more severe symptoms.
(3) PHQ-9 [23], 24]: It includes nine items (0–27); higher scores indicate severer symptoms.
(4) SF-12 [17], 25]: This scale assessed health-related quality of life, including Physical Component Summary (PCS) and Mental Component Summary (MCS) (max 100 each). Higher scores indicate better quality of life.
(5) MoCA-C [26], 27]: Used to evaluate overall cognitive function, including seven dimensions: visual space/executive function, naming, attention, language, abstract thinking, delayed recall, and orientation. The total score is 30 points, with <26 points indicating cognitive impairment, and higher scores indicating better cognitive function.

### Statistical analysis

Data analysis was conducted with SPSS 25.0. The Kolmogorov-Smirnov test was used to examine normality. Normally distributed data were shown as mean ± SD and compared by independent-samples t-test; non-normal data as M (Q1, Q3) with Mann-Whitney U test. Categorical data were presented as n (%) and analyzed via χ^2^ test. Spearman rank correlation coefficient was used for correlation analysis. Multivariate logistic regression was applied to explore risk factors, with p<0.05 considered statistically significant. Due to the multiple comparisons of multiple scales involved in this study, Bonferroni correction was used to control the cumulative risk of Class I errors. The corrected inspection level is set at α’=0.05/number of comparisons. If the p-value is still less than the corresponding α′after correction, it is considered statistically significant.

## Results

### Comparison of patient baseline data

Ultimately, 120 elderly patients were recruited in the present study, and 50 of them were assigned to the SD group, with an incidence rate of 41.7 %, and 70 cases were included in the non-sleep disturbance (NSD) group, accounting for 58.3 %. Univariate analysis (Table 1) demonstrated that the proportions of patients with chronic pain, subjective loneliness, and long-term use of sedative-hypnotic drugs were markedly higher in the SD group than in the NSD group, showing statistically significant differences (p<0.05). However, no statistically significant intergroup differences were observed in sex, BMI, marital status, educational level, and the proportion of patients complicated with ≥2 underlying diseases (p>0.05).

**Table 1:** Baseline characteristics [mean ± SD, n (%)].

Variables	SD group (n=50)	NSD group (n=70)	χ^2^/t	p-Value
Age, years	73.5 ± 5.8	72.0 ± 6.5	1.293	0.199
≥75	20 (40.0)	20 (28.0)	1.714	0.190
Gender				
Male	22 (44.0)	36 (51.4)		
Female	28 (56.0)	34 (48.6)	0.645	0.422
BMI, kg/m^2^	23.2 ± 3.4	23.6 ± 2.9	0.811	0.419
≥23.5	22 (41.5)	36 (53.7)	1.377	0.214
Marital status				
Married	33 (66.0)	52 (74.3)		
Widowed/Divorced/Unmarried	17 (34.0)	18 (25.7)	0.969	0.325
Educational level				
Junior high school or below	32 (64.0)	42 (60.0)		
Senior high school or above	18 (36.0)	28 (40.0)	0.197	0.657
Cardiovascular disease	14 (28.0)	23 (32.9)	0.323	0.570
Diabetes	17 (34.0)	23 (32.9)	0.017	0.896
Chronic pain	23 (46.0)	17 (24.3)	6.189	0.013
Comorbidities				
<2	15 (30.0)	31 (44.3)		
≥2	35 (70.0)	39 (55.7)	2.518	0.113
Long-term use of sedative-hypnotics				
Yes	20 (40.0)	16 (22.9)		
No	30 (60.0)	54 (77.1)	4.082	0.043
Subjective loneliness				
Present	27 (54.0)	25 (35.7)		
Absent	23 (46.0)	45 (64.3)	3.972	0.046

BMI, body mass index. The same below.

### Sleep quality analysis

Analysis of the seven domain scores of the PSQI (Table 2) revealed that scores in all seven dimensions, were significantly higher in the SD group than in the NSD group (p<0.001), which fully indicates that sleep disturbance in this population presents comprehensive impairments across all domains.

**Table 2:** Comparison of PSQI scores in various dimensions [M(Q1, Q3)].

Variables	SD group (n=50)	NSD group (n=70)	Z	p-Value
Subjective sleep quality	2 (1, 2)	1 (0, 1)	5.946	<0.001
Sleep latency	2 (2, 3)	1 (0, 1)	6.979	<0.001
Sleep duration	2 (1, 2)	1 (0, 1)	7.093	<0.001
Sleep efficiency	2 (1, 2)	1 (0, 1)	5.770	<0.001
Sleep disturbances	2 (1, 2)	1 (0, 1)	6.803	<0.001
Use of hypnotic medication	1 (0, 2)	0 (0, 1)	4.344	<0.001
Daytime dysfunction	2 (1, 2)	1 (0, 1)	6.212	<0.001
Total PSQI score	12 (10, 14)	5 (4, 5)	9.315	<0.001

PSQI, pittsburgh sleep quality index. The normality test (Kolmogorov Smirnov) showed that the total score of PSQI and each dimension did not follow a normal distribution (p<0.05), so Mann Whitney U test was used. Bonferroni correction for eight comparisons (7 dimensions + total score) sets significance threshold at p<0.006. All comparisons remained significant after correction.

### Comparison of psychological state, quality of life and cognitive function

A retrospective analysis of psychological, quality of life and cognitive function assessments (Table 3) showed that HADS-anxiety scores, HADS-depression scores, and PHQ-9 scores in the SD group were markedly higher than those in the NSD group (p<0.001), suggesting that patients with sleep disturbance presented more severe anxiety and depression. Regarding quality of life, both PCS and MCS scores of the SF-12 in the SD group were notably lower than those in the NSD group (p<0.001), indicating poorer quality of life among patients with sleep disturbance. In terms of overall cognitive function, the MoCA-C score of the SD group was significantly lower than that of the NSD group (p<0.001), indicating that patients with sleep disorders have lower overall cognitive function.

**Table 3:** Comparison of HADS, PHQ-9, and SF-12 scores (mean ± SD).

Variables	SD group (n=50)	NSD group (n=70)	t	p-Value
HADS: Anxiety	8.8 ± 3.3	5.5 ± 2.8	5.969	<0.001
HADS: Depression	9.0 ± 3.5	5.8 ± 3.1	5.376	<0.001
PHQ-9	11.4 ± 4.4	6.2 ± 3.3	7.343	<0.001
SF-12: PCS	41.8 ± 3.3	49.0 ± 3.7	10.823	<0.001
SF-12: MCS	44.7 ± 3.8	52.8 ± 3.3	12.571	<0.001
MoCA-C	20.4 ± 3.3	24.0 ± 3.5	5.717	<0.001

HADS, hospital anxiety and depression scale; PHQ-9, patient health questionnaire-9; SF-12, 12-item short-form health survey; PCS, physical component summary; MCS, mental component summary; MoCA-C, Chinese montreal cognitive assessment. The same below. Between-group comparisons: independent t-test. Bonferroni correction for seven comparisons sets significance threshold at p<0.007. All comparisons remained significant after correction.

### Correlation analysis of sleep quality, psychological state, quality of life and cognitive function

Correlation analysis (Table 4) showed that PSQI total score was positively correlated with HADS-anxiety, HADS-depression and PHQ-9 scores (r=0.413, 0.422, 0.514; all p<0.001), and negatively correlated with SF-12 PCS, MCS and MoCA scores (r=−0.638, −0.683, −0.476; p<0.001). Poorer sleep quality was linked to more severe anxiety, depression, lower quality of life and the overall cognitive function.

**Table 4:** Correlation analysis.

Variables	ρ	p-Value
HADS: Anxiety	0.413	<0.001
HADS: Depression	0.422	<0.001
PHQ-9	0.514	<0.001
SF-12: PCS	−0.638	<0.001
SF-12: MCS	−0.683	<0.001
MoCA-C	−0.476	<0.001

All variables were non-normally distributed (Kolmogorov-Smirnov, p<0.05). Spearman rank correlation coefficient (ρ) is reported.

### Univariate logistic regression analysis of sleep disorders affecting the elderly

To preliminarily screen potential risk factors for sleep disturbance in elderly individuals, univariate Logistic regression analysis was performed with sleep disturbance as the dependent variable (0=no, 1=yes), and demographic and clinical characteristics in Table 1 as independent variables (Table 5). The results indicated that chronic pain, long-term use of sedative-hypnotic drugs, and loneliness were potential risk factors (OR=2.275–2.638, p<0.05).

**Table 5:** Univariate logistic regression analysis.

						95 % CI for OR	
Variables	B	SE	Wald	P	OR	Lower	Upper

Age 75	0.354	0.389	0.825	0.364	1.424	0.664	3.055
Male	−0.355	0.370	0.924	0.336	0.701	0.340	1.447
BMI 23.5	−0.492	0.371	1.761	0.185	0.611	0.295	1.265
Married	−0.414	0.404	1.051	0.305	0.661	0.300	1.458
Senior high school or above	−0.189	0.380	0.248	0.619	0.828	0.393	1.742
Cardiovascular disease	0.104	0.397	0.069	0.793	1.110	0.509	2.418
Diabetes	0.202	0.389	0.27	0.603	1.224	0.571	2.624
Chronic pain	0.970	0.398	5.945	0.015	2.638	1.210	5.753
Comorbidities 2	0.334	0.381	0.765	0.382	1.396	0.661	2.948
Long-term use of sedative-hypnotics	0.822	0.406	4.101	0.043	2.275	1.027	5.041
Subjective loneliness	0.838	0.377	4.932	0.026	2.312	1.103	4.842

### Multivariate logistic regression analysis of sleep disorders affecting the elderly

The variables (chronic pain, long-term use of sedatives, subjective loneliness) with p<0.10 in the univariate analysis were all included in the multifactor logistic regression model for analysis (Table 6), and the key variables, diabetes and cardiovascular disease, were also included. The results showed that chronic pain (OR=3.580, 95 % CI: 1.444–8.879, p=0.006), long-term use of sedative hypnotic drugs (OR=2.860, 95 % CI: 1.136–7.200, p=0.026), and psychological loneliness (OR=5.261, 95 % CI: 2.006–13.798, p=0.001) were independent risk factors for the development of sleep disorders in the elderly. This means that patients with chronic pain, long-term use of sedative hypnotic drugs, and psychological loneliness have an increased incidence of sleep disorders (odds) by 258.0 , 186.0, and 426.1 %, respectively.

**Table 6:** Multivariate logistic regression analysis.

						95 % CI for OR	
Variables	B	SE	Wald	P	OR	Lower	Upper

Cardiovascular disease	0.382	0.459	0.694	0.405	1.465	0.596	3.600
Diabetes	0.407	0.458	0.789	0.374	1.502	0.612	3.687
Chronic pain	1.275	0.463	7.576	0.006	3.580	1.444	8.879
Long-term use of sedative-hypnotics	1.051	0.471	4.973	0.026	2.860	1.136	7.200
Subjective loneliness	1.660	0.492	11.391	0.001	5.261	2.006	13.798
Constant	−1.982	0.504	15.452	<0.001	0.138		

Hosmer and lemeshow test χ^2^=9.159, df=7, p=0.241, Nagelkerke R^2^=0.225.

### Decomposition analysis of quality of life under different sleep disorder states

To precisely characterize the specific pathways through which sleep disturbance impairs quality of life, this study retrospectively extracted scores on eight specific domains of the SF-12 scale. The analysis revealed that scores on all eight domains were significantly lower in the SD group than in the NSD group, with particularly severe impairments observed in VT and MH. Details are shown in Table 7.

**Table 7:** Comparison of the eight dimensions of SF-12 (mean ± SD).

SF-12	SD group (n=50)	NSD group (n=70)	t	p-Value
GH	43.8 ± 10.1	48.8 ± 10.3	2.650	0.009
PF	47.9 ± 12.2	55.4 ± 11.4	3.486	0.003
RP	45.8 ± 10.6	50.9 ± 13.3	2.270	0.025
BP	42.3 ± 13.9	48.9 ± 13.6	2.583	0.011
VT	38.7 ± 9.7	49.5 ± 9.6	6.064	<0.001
SF	44.2 ± 12.3	52.4 ± 11.7	3.713	<0.001
RE	41.2 ± 11.7	49.1 ± 10.7	3.827	<0.001
MH	45.1 ± 9.4	54.4 ± 8.4	5.666	<0.001

GH, general health; PF, physical functioning; RP, role physical; BP, bodily pain; VT, vitality; SF, social functioning; RE, role emotional; MH, mental health. Between-group comparisons: independent t-test. Bonferroni correction for eight comparisons sets significance threshold at p<0.006. All comparisons remained significant after correction.

## Discussion

This retrospective study collected and rigorously screened detailed clinical data and follow-up scale assessments of 120 elderly patients from 2020 to 2025, and comprehensively investigated the epidemiological characteristics of sleep disturbance in the elderly, as well as its multi-dimensional associations with anxiety, depression, quality of life, and overall cognitive function. The results clearly demonstrated that the prevalence of sleep disturbance in this elderly cohort reached 41.7 %. Anxiety and depression scores were significantly higher in the SD group than in the NSD group, with marked decreases in both physical and mental quality of life. Pearson correlation analysis further confirmed strong positive correlations between total PSQI scores and negative emotions (HADS, PHQ-9), as well as negative correlations with quality of life (SF-12) and cognitive function (MoCA). In addition, multivariate Logistic regression analysis identified chronic pain, long-term use of sedative-hypnotic drugs and subjective loneliness as independent risk factors for sleep disturbance in the elderly. These findings strongly support the preliminary clinical hypothesis. Moreover, they offer insights for developing targeted comprehensive management strategies.

Sleep disturbance prevalence of 41.7 % identified in this retrospective dataset is highly consistent with conclusions drawn from recent global multicenter epidemiological investigations. As studied by Patel et al. [28], the overall prevalence of insomnia and obstructive sleep apnea syndrome among the elderly population in Western developed countries ranges from 40 to 50 %. Such high consistency across different races and regions strongly suggests that sleep disturbance in old age is underpinned by profound biological inevitability. In the multivariate regression model of this study, chronic pain was verified as an independent risk factor for sleep disorders (OR=3.580). Long-term administration of sedative-hypnotic agents not only fails to enhance overall sleep quality but also serves as an independent risk factor for the persistence or worsening of sleep disorders (OR=2.860). This seemingly paradoxical result actually reveals the substantial hazards of polypharmacy in the elderly. Long-term administration of benzodiazepines or Z-drugs can induce receptor tolerance and psychological dependence [29]. When the dosage cannot be increased further, patients may experience severe rebound insomnia. Moreover, chronic use alters sleep architecture (e.g., suppressing REM and deep slow-wave sleep), leading to non-restorative sleep, daytime somnolence, and cognitive hangover effects [30]. The strong effect of chronic pain confirms the bidirectional mutual promotion mechanism between pain and sleep. Nocturnal pain can directly interrupt sleep continuity and induce pain sensitization. The effect of psychological loneliness (OR=5.261) is most prominent, indicating that social isolation severely hinders the transition from bedtime to a relaxed state by activating the sympathetic nervous system and increasing cortisol levels. Long term use of sedatives and hypnotics remains an independent risk factor, revealing the clinical dilemma between drug tolerance and abnormal insomnia. The coexistence of the three suggests that sleep management for the elderly requires comprehensive intervention from three aspects: pain relief, social and psychological support, and reasonable reduction and cessation of medication.

Data from Table 3 and Table 4 in this study verified the tight interdependent relationship between sleep disturbance and anxiety-depression with highly significant statistical differences. HADS and PHQ-9 scores in the SD group even reached the clinical intervention threshold for mild to moderate depression and anxiety. An extremely complex bidirectional interactive network of neuroendocrine and neuroimmunological mechanisms underlies this phenomenon [31]. From the perspective of the HPA axis, sleep acts as a key recovery phase against stress accumulation during wakefulness. Previous basic and clinical research (e.g., Irwin 2019) confirmed that long-term sleep deprivation impairs the feedback inhibition of the HPA axis, resulting in pathological nocturnal elevations of serum cortisol and adrenocorticotropic hormone [32]. Sustained hypercortisolemia exerts strong neurotoxicity, which directly suppresses neurogenesis and triggers synaptic atrophy in the dentate gyrus of the hippocampus, a core region for emotional regulation and memory, further inducing depression-like behaviors and anxiety symptoms [33]. The central and peripheral neuroinflammation hypothesis has gained extensive attention in the past five years. Chronic insomnia is recognized as a chronic systemic physical stress. Studies have revealed that weakened rest-activity rhythms, an indicator of sleep disturbance, are significantly correlated with elevated inflammatory markers in elderly men [34]. These inflammatory cytokines can activate microglia via permeable regions of the blood-brain barrier and elicit CNS inflammation. This disturbs the tryptophan-kynurenine pathway, depletes serotonin, and ultimately precipitates depressive episodes [35]. Conversely, depression and anxiety per se disrupt sleep via abnormal hyperactivity of the default mode network. Hyperarousal and rumination in anxious states prevent the cerebral cortex from shifting to a synchronized low-frequency electroencephalogram pattern before sleep, directly prolonging sleep latency and markedly lowering sleep efficiency as displayed in Table 2 [36]. This establishes a vicious cycle: insomnia – inflammation/endocrine disorders – aggravated anxiety and depression – hyperarousal - exacerbated insomnia [37].

The detailed breakdown of the SF-12 in Table 7 reveals that sleep disturbance inflicts comprehensive damage on quality of life. Notably, the SD group exhibited extremely poor scores in the VT and MH dimensions (p<0.001). The mechanisms underlying impaired quality of life are multi-dimensional. With respect to PF and RP, the absence of high-quality deep sleep leads to a sharp decline in growth hormone secretion in elderly individuals, inhibiting muscle protein synthesis and readily inducing or exacerbating sarcopenia and frailty syndrome [38]. The decline in muscle strength, combined with the residual effects of sedative-hypnotic drugs, places this elderly population at high risk of falls and fractures, directly undermining their physiological capacity for independent living. In the BP dimension, sleep deprivation has been firmly established to reduce the brain’s pain tolerance threshold, namely pain sensitization, which subjectively amplifies mild joint and bone pain and further limits mobility [39]. For SF and RE, severe daytime sleepiness and low mood reduce patients’ willingness to participate in family gatherings or community activities, resulting in a rapid shrinkage of their social support networks. This also interacts reciprocally with subjective loneliness (OR=5.261) identified in this study. Particularly during the 2020–2025 data collection period, which overlapped with the COVID-19 pandemic, public health restrictions and social distancing policies led to widespread social isolation among the elderly. As a profound psychological stressor, loneliness activates the sympathetic nervous system and severely impedes the transition to a deeply relaxed sleep state [40].

## Research limitations

Although this retrospective analysis with rigorous methodology verified the study hypotheses, several limitations should be acknowledged. First, as a single-center retrospective observational study, the sample size (n=120) was relatively limited, which may lead to insufficient sample representativeness due to hospital-based selection bias, resulting in some secondary risk factors failing to reach statistical significance. Second, although logistic regression was applied to adjust for several confounders, the retrospective design is inherently weaker in verifying causal relationships than prospective randomized controlled trials and large-scale longitudinal cohort studies. Third, the evaluation of sleep quality relied entirely on subjective scales (PSQI) without corroboration from objective physiological indicators such as polysomnography or actigraphy, which may introduce certain recall bias. Fourth, this hospital-based psychiatric sample limits generalizability to community-dwelling older adults. The observed prevalence and associations may be inflated. Community-based validation is needed.

## Conclusions

In summary, this retrospective study of 120 elderly patients draws the following clear conclusions: the PSQI score is significantly positively correlated with the severity of negative psychological symptoms such as anxiety and depression. Chronic pain, long-term use of irrational sedative hypnotic drugs, and psychological loneliness are independent risk factors for the development of sleep disorders in the elderly. In future clinical practice and public health management, medical workers must be highly vigilant about the sleep, emotions, and quality of life of elderly patients, and actively promote an individualized comprehensive management model integrating psychological counseling, lifestyle intervention, and rational drug use, so as to truly achieve the ultimate goal of healthy aging.

## References

[1] Ohayon MM, Carskadon MA, Guilleminault C, Vitiello MV (2004). Meta-analysis of quantitative sleep parameters from childhood to old age in healthy individuals: developing normative sleep values across the human lifespan. Sleep.

[2] Li J, Vitiello MV, Gooneratne NS (2018). Sleep in normal aging. Sleep Med Clin.

[3] Mander BA, Winer JR, Walker MP (2017). Sleep and human aging. Neuron.

[4] Miner B, Kryger MH (2020). Sleep in the aging population. Sleep Med Clin.

[5] Gulia KK, Kumar VM (2018). Sleep disorders in the elderly: a growing challenge. Psychogeriatrics.

[6] Bertollo AG, Puntel CF, Albuquerque MFPA, Narzetti R, Ignácio ZM (2025). The impact of lifestyle on depression and anxiety in older adults. Neurosci Res Clin Pract.

[7] Sindi S, Pérez LM, Vetrano DL, Triolo F, Kåreholt I, Sjöberg L (2020). Sleep disturbances and the speed of multimorbidity development in old age: results from a longitudinal population-based study. BMC Med.

[8] Gulali AMP, Tel BMA, Bilgin S, Basaran E, Khalid A, Cimenden E (2025). The relationship between frailty, hemoglobin-albumin-iymphocyte-platelet (HALP) score, and systemic inflammatory index (SII) in elderly type 2 diabetic patients. J Endocr Syst Res.

[9] Xu H, Li X, Liu C (2025). Bridge symptoms of depression and anxiety among older adults in China: a longitudinal network comparison by living arrangements. Front Psychiatr.

[10] Hood S, Amir S (2017). The aging clock: circadian rhythms and later life. J Clin Invest.

[11] Suzuki K, Miyamoto M, Hirata K (2017). Sleep disorders in the elderly: diagnosis and management. J Gen Fam Med.

[12] Reynolds CF, Jeste DV, Sachdev PS, Blazer DG (2022). Mental health care for older adults: recent advances and new directions in clinical practice and research. World Psychiatry.

[13] Irwin MR (2019). Sleep and inflammation: partners in sickness and in health. Nat Rev Immunol.

[14] Wen B, Zhang G, Zhan C, Chen C, Yi H (2025). The 2024 revision of the declaration of Helsinki: a modern ethical framework for medical research. Postgrad Med J.

[15] Kim N, Fischer AH, Dyring-Andersen B, Rosner B, Okoye GA (2017). Research techniques made simple: choosing appropriate statistical methods for clinical research. J Invest Dermatol.

[16] Hwang G, Cho YH, Kim EJ, Woang JW, Hong CH, Roh HW (2022). Differential effects of sleep disturbance and malnutrition on late-life depression among community-dwelling older adults. Front Psychiatr.

[17] Shou J, Ren L, Wang H, Yan F, Cao X, Wang H (2016). Reliability and validity of 12-item short-form health survey (SF-12) for the health status of Chinese community elderly population in Xujiahui district of Shanghai. Aging Clin Exp Res.

[18] Buysse DJ, Reynolds CF, Monk TH, Berman SR, Kupfer DJ (1989). The Pittsburgh sleep quality index: a new instrument for psychiatric practice and research. Psychiatry Res.

[19] Peerbhay A, Miseer P, Lowton K (2025). Assessing sleep using the Pittsburgh sleep quality index (PSQI) among comorbid HIV and psychiatric outpatients. S Afr J Psychiatr.

[20] Tsai PS, Wang SY, Wang MY, Su CT, Yang TT, Huang CJ (2005). Psychometric evaluation of the Chinese version of the Pittsburgh sleep quality index (CPSQI) in primary insomnia and control subjects. Qual Life Res.

[21] Zigmond AS, Snaith RP (1983). The hospital anxiety and depression scale. Acta Psychiatr Scand.

[22] Yang Y, Ding R, Hu D, Zhang F, Sheng L (2014). Reliability and validity of a Chinese version of the HADS for screening depression and anxiety in psycho-cardiological outpatients. Compr Psychiatry.

[23] Kroenke K, Spitzer RL, Williams JB (2001). The PHQ-9: validity of a brief depression severity measure. J Gen Intern Med.

[24] Wang W, Bian Q, Zhao Y, Li X, Wang W, Du J (2014). Reliability and validity of the Chinese version of the patient health questionnaire (PHQ-9) in the general population. Gen Hosp Psychiatry.

[25] Ware J, Kosinski M, Keller SD (1996). A 12-Item short-form health survey: construction of scales and preliminary tests of reliability and validity. Med Care.

[26] Nasreddine ZS, Phillips NA, Bédirian V, Charbonneau S, Whitehead V, Collin I (2005). The Montreal cognitive assessment, MoCA: a brief screening tool for mild cognitive impairment. J Am Geriatr Soc.

[27] Hu JB, Zhou WH, Hu SH, Huang ML, Wei N, Qi HL (2013). Cross-cultural difference and validation of the Chinese version of Montreal cognitive assessment in older adults residing in Eeastern China: preliminary findings. Arch Gerontol Geriatr.

[28] Patel D, Steinberg J, Patel P (2018). Insomnia in the elderly: a review. J Clin Sleep Med.

[29] Maust DT, Blow FC, Wiechers IR, Kales HC, Marcus SC (2017). National trends in antidepressant, benzodiazepine, and other sedative-hypnotic treatment of older adults in psychiatric and primary care. J Clin Psychiatry.

[30] Riemann D, Espie CA, Altena E, Arnardottir ES, Baglioni C, Bassetti CLA (2023). The European insomnia guideline: an update on the diagnosis and treatment of insomnia 2023. J Sleep Res.

[31] Hertenstein E, Feige B, Gmeiner T, Kienzler C, Spiegelhalder K, Johann A (2019). Insomnia as a predictor of mental disorders: a systematic review and meta-analysis. Sleep Med Rev.

[32] Irwin MR, Piber D (2018). Insomnia and inflammation: a two hit model of depression risk and prevention. World Psychiatry.

[33] McEwen BS, Nasca C, Gray JD (2016). Stress effects on neuronal structure: hippocampus, amygdala, and prefrontal cortex. Neuropsychopharmacology.

[34] Xiao Q, Qian J, Evans DS, Redline S, Lane NE, Ancoli-Israel S (2022). Cross-sectional and prospective associations of rest-activity rhythms with circulating inflammatory markers in older men. J Gerontol A Biol Sci Med Sci.

[35] Dantzer R, O’Connor JC, Freund GG, Johnson RW, Kelley KW (2008). From inflammation to sickness and depression: when the immune system subjugates the brain. Nat Rev Neurosci.

[36] Riemann D, Spiegelhalder K, Feige B, Voderholzer U, Berger M, Perlis M (2010). The hyperarousal model of insomnia: a review of the concept and its evidence. Sleep Med Rev.

[37] Freeman D, Sheaves B, Goodwin GM, Yu LM, Nickless A, Harrison PJ (2017). The effects of improving sleep on mental health (OASIS): a randomised controlled trial with mediation analysis. Lancet Psychiatry.

[38] Piovezan RD, Abucham J, Dos Santos RV, Mello MT, Tufik S, Poyares D (2015). The impact of sleep on age-related sarcopenia: possible connections and clinical implications. Ageing Res Rev.

[39] Finan PH, Goodin BR, Smith MT (2013). The association of sleep and pain: an update and a path forward. J Pain.

[40] Cacioppo JT, Hawkley LC, Berntson GG, Ernst JM, Gibbs AC, Stickgold R (2002). Do lonely days invade the nights? potential social modulation of sleep efficiency. Psychol Sci.

